# Universal Micromachining Platform and Basic Technologies for the Manufacture and Marking of Microphysiological Systems

**DOI:** 10.3390/mi8080246

**Published:** 2017-08-11

**Authors:** Katja Günther, Frank Sonntag, Elmar Moritzer, Andrè Hirsch, Udo Klotzbach, Andrés Fabián Lasagni

**Affiliations:** 1 Institute of Manufacturing Technology, Technische Universität Dresden, George-Baehr-Str.1, 01069 Dresden, Germany; andres_fabian.lasagni@tu-dresden.de; 2 Fraunhofer Institute for Material and Beam Technology IWS, Winterbergstrasse 28, 01277 Dresden, Germany; frank.sonntag@iws.fraunhofer.de (F.S.); udo.klotzbach@iws.fraunhofer.de (U.K.); 3 Kunststofftechnik Paderborn, Universität Paderborn University, Warburger Straße 100, 33098 Paderborn, Germany; elmar.moritzer@ktp.upb.de (E.M.); andre.hirsch@ktp.uni-paderborn.de (A.H.)

**Keywords:** microfluidic, lab-on-a-chip, 3D printing, direct laser writing, direct laser interference patterning

## Abstract

Micro Physiological Systems (MPS), also known as Multi-Organ-Chip, Organ-on-a-Chip, or Body-on-a-Chip, are advanced microfluidic systems that allow the cultivation of different types of cells and tissue in just one common circuit. Furthermore, they thus can also adjust the interaction of these different tissues. Perspectival MPS will replace animal testing. For fast and flexible manufacturing and marking of MPS, a concept for a universal micromachining platform has been developed which provides the following latest key technologies: laser micro cutting of polymer foils, laser micro- and sub-micro-structuring of polymer foils, 3D printing of polymer components as well as optical inspection and online process control. The combination of different laser sources, processing optics, inspection systems, and print heads on multiple axes allows the change and exactly positioning to the workpiece during the process. Therewith, the realization of MPS including 3D printed components as well as direct laser interference patterned surfaces for well-defined cell adhesion and product protection is possible. Additional basic technologies for the generation of periodical line-like structures at polycarbonate foils using special Direct Laser Interference Patterning (DLIP) optics as well as for the 3D printing of fluid-tight cell culture reservoirs made of Acrylonitrile Butadiene Styrene directly onto polycarbonate microfluidics were established. A first prototype of the universal micromachining platform combining different lasers with Direct Laser Writing and DLIP is shown. With this laser micro cutting as well as laser micro-structuring of polycarbonate (PC) foils and therewith functionalization for MPS application could be successfully demonstrated.

## 1. Introduction

Lab-on-a-Chip-Systems are utilized for medicine and biotechnology applications. The field reaches from synthesis of active pharmaceutical ingredients up to the detection of specific biomarkers and the cultivation of human cells and human tissues for substance testing and regenerative medicine. The latter has a substantial interest because micro-perfusion-systems enable a much higher cell concentration and replicate the in vivo situation much better than statically cell culture systems [1]. Due to that Micro Physiological Systems (MPS), also known as Multi-Organ-Chip, Organ-on-a-Chip, or Body-on-a-Chip, have benefit. MPS are miniaturized, chip-sized platforms designed to emulate the physical environment for in vitro cell cultures. They can be used as cellularized organoid systems to study cellular processes like migration, regeneration, or proliferation. They are also able to simulate the interaction of different organs as well as pharmacokinetics. That means the adsorption, distribution, metabolism, and excretion of drugs and metabolites. MPS show wide application possibilities for various cell types and organ models [2]. Perspectival MPS will replace animal testing. This is necessary, since the results of animal testing can be transferred more and more limited to humans in the pharmaceutical active ingredient testing [3]. In MPS, various organoids like skin [4], liver [5,6], endothelium [7], hair follicle [8], intestine [5], and kidney [4] were successfully cultivated. Furthermore, the platform offers interfaces for the integration of scaffolds and artificial blood vessels, for example hollow fibers [9]. It is possible to continuously monitor vitality and other process parameters such as, the oxygen concentration [10], for example.

However, MPS can be produced combining a layer-by-layer manufacturing technology of laser-cut [9,11] and structured [12,13,14,15,16] polymer foils and 3D printing of application-specific cell culture modules and reservoirs [17]. For fast and flexible manufacturing and marking of tailor-made MPS a universal micromachining platform is needed which provides the following latest key technologies:

- laser micro cutting of polymer foils [9,11],
- laser micro- und sub-micro-structuring of polymer foils [12,13,14,15,16],
- 3D printing of polymer components [17],
- Optical inspection and online process control.

Therewith, the realization of MPS including 3D printed components, as well as direct laser interference patterned surfaces for well-defined cell adhesion and product protection is possible.

## 2. Materials and Methods

### 2.1. Micro Physiological Systems

The MPS is comprised of a basic chip, application-specific cell culture modules, and reservoirs. The basic chip consists of a multilayer stack of micro structured, functionalized, and bonded polymer foils. The multilayer technology also enables the realization of pneumatically driven pumps and valves. By applying an overpressure to a thin polymer membrane, it bulges and displaces the liquid in the chamber below. If, on the other hand, a vacuum is applied, the membrane retracts and thus increases the volume of the chamber. The connection of pump and valve chambers to a peristaltic pump and suitable control allows the directed transport of liquids. The use of highly transparent polymers allows optical access for non-invasive online monitoring. By using different laser technologies, well-defined surface properties (e.g., topography, chemistry, and product protection) can be realized locally. The basic chips are manufactured with an established closed technology chain, developed at Fraunhofer IWS. In the first step, the basic chip, which is to be realized, is constructively split into individual layers, which are later formed in each case by a separate foil. In the second step, a material with the desired properties (hydrophilic, hydrophobic, transparent, gas-permeable, etc.) is selected from the functional boundary conditions for each layer. In the third step, the foils are cut by means of laser micro-material processing, structured on both sides and optionally functionalized [9,11,13,18]. In the fourth and final step, the individual foils are laminated together into a multilayer system using different technologies (thermal bonding, bonding, welding, etc.). 

The application-specific reservoirs and cell culture segments are prepared by means of generative production processes, e.g., 3D printing or selective laser sintering of polymers [17].

The basic chip, with the exception of the membrane, is made out of polycarbonate (PC) foils (product number ST0262951, thickness: 250 µm, Modulor GmbH, Berlin, Germany).

The cell culture segments were made of Acrylonitrile Butadiene Styrene (ABS, Terluran GP35) using the Arburg freeformer. 

### 2.2. Laser Treatment of Polycarbonate Foils 

The used PC foil has an average surface roughness of 18 nm. The laser micro structuring methods, direct laser writing (DLW) and direct laser interference patterning (DLIP) were utilized. Functionalized micro structured surfaces were fabricated with three different short-pulse laser-systems:

- Fuego, Time-Bandwidth Products, pulse duration (FWHM) <10 ps, wavelength: 355 nm, maximum repetition rate: 200 kHz, maximum pulse energy: 50 µJ;
- Quanta-Ray Pro290, Spectra Physics, pulse duration (FWHM) <10 ns, wavelength: 266 nm, maximum repetition rate: 10 Hz, maximum pulse energy: up to 180 mJ;
- TECH-263 advanced, Laser Export Co. Ltd., Pulse duration (FWHM) <3 ns, wavelength: 263 nm, maximum repetition rate: 4 kHz, pulse energy up to 50 µJ.

Depending on the structure size, different optical setups were used for the DLW process. For large structures a static laser beam is focused onto the substrate surface by means of a lens. Structuring can be realized moving the substrate relative to the static beam (DLW-module). For small structures a galvanometer scanning head can be used (Scanner-module). In this setup, the substrate is fixed and the laser beam is deflected relative to the surface. The details were described elsewhere [11]. 

However, during the DLIP process, two or more laser beams are superimposed onto the substrate surface. This produces an interference volume with which different periodic structures (lines, holes, crossline) are generated [12,13,19,20]. In general, the number of used laser beams as well as their individual phase and polarization control the shape of the interference pattern. Differently, the structure period can be changed by varying the utilized laser wavelength and the intercepting angle of the interfering beams. Depending on the wavelength and material used, structure periods between ~150 nm and ~30 μm have been already reported [19]. However, the size of the achievable structural parameters do not only depend on the spatial period of the interference pattern but on the kind of interaction between the laser light and the material (e.g., melting, evaporation, etc.). In the case of processing of polymer substrates, spatial periods even in the sub-micrometer range could be obtained due to the very short thermal diffusion lengths compared to metals, as it has been shown for polycarbonate [14], polyurethane [15], or poly (styrene-co-acrylonitrile) [16].

Due to the above mentioned characteristics, significantly shorter processing times are required for the DLIP-technology compared to DLW, especially to fabricate patterns with small feature sizes [14]. Depending on the desired spatial period, different optical setups can be used. For long periods, we used a setup of mirrors and beam splitters that permit to change incident angle even to very small values (<1°) [13]. For small spatial periods, a combination of a Diffractive Optical Element (DOE), a lens and a prism was used as recently reported [20]. The structured substrates were characterized using a confocal microscope ( Sensofar S Neox, Barcelona, Spain) with a magnification of 20×, 50×, and 150× and maximum lateral and vertical resolutions of 140 and 2 nm at 150×, respectively. 

### 2.3. Plastic Freeforming

The capability of 3D printing technologies for direct production of complex 3D polymer components in a single step has recently attracted an ever increasing interest within the field of microfluidics [17,21,22,23]. 

Plastic Freeforming (PF) is an additive manufacturing process to produce three-dimensional plastic parts based on 3D CAD data by applying plastic droplets layer by layer. The freeformer was developed to transform the PF-process into a viable industrial process. At first qualified standard granulates are melted as in the injection molding process to generate a defined dosage volume in the screw chamber. The axial movement of the plasticizing screw displaces the plastic melt into the discharge unit. The main component of the discharge unit is a piezoelectric, high frequency driven nozzle closure. Due to the rapid opening and closing of the nozzle up to 140 tiny droplets with a diameter of between 0.2 and 0.3 mm can be produced per second. A movable three-axis part carrier ensures that the single droplets are accurately positioned on the component structure, whereby the specific deposition strategy can be adjusted by varying the process parameters. The freeformer is equipped with two plasticizing units and two discharge units to realize multi-material combinations in one process step. The open machine controller system enables all processing parameters to be freely programmable and permits that the processes can be individually optimized for different materials. In principle, the freeformer can be operated with any thermoplastic material, including biocompatible polymers. For complex geometries water-soluble support material, based on polyvinylpyrrolidone, is used.

## 3. Results and Discussion

### 3.1. Conception for a Universal Micromachining Platform

For manufacturing and marking of MPS, a universal micromachining platform has to provide the following latest key technologies:

- Laser micro cutting of polymer foils,
- Laser micro- und sub-micro-structuring of polymer foils,
- 3D printing of polymer components as well as,
- Optical inspection and online process control.

Different laser sources, processing optics, inspection systems, and print heads are required for the individual processes. Different axes allow the change of the modules and exactly positioning of the workpiece during the process. 

**Figure 1:**
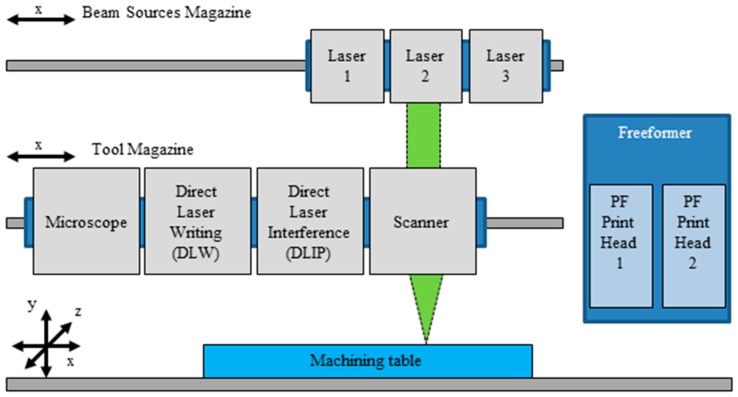
Conception of the universal micromachining platform for manufacturing and marking of Micro Physiological Systems (MPS) using latest key technologies: laser micro cutting of polymer foils, laser micro- and sub-micro-structuring (direct laser writing (DLW), Scanner and Direct Laser Interference Patterning-module (DLIP-module) of polymer foils, 3D printing of polymer components as well as optical inspection and online process control.

Figure 1 shows the conception of the universal micromachining platform. The machining table with the workpiece holder is mounted on a three-axis positioning system. On the left side, two linear axes are arranged horizontally above the worktable. The various tools (processing optics, microscope, print heads) are mounted on the lower axis (tool magazine), and the different laser sources are mounted on the upper axis (beam source magazine). By appropriately aligning the tool and beam source magazine, different processing optics (direct laser writing, mask projection, two-beam and multi-beam interference, scanner, etc.) can be combined with different beam sources (lasers). The DLW-module allows structuring of large areas (determined by range of the axes) using a relative movement between the substrate and the incident laser beam. Using the scanner-module working field and spot diameter are determined by the objective. With DLIP-module, different kinds of periodic structures can be realized.

On the right side, the freeformer with the two PF print heads is mounted. The corresponding positioning of the machining table for the selected tool is carried out for processing.

### 3.2. Demonstrator of the Universal Micromachining Platform

The current function pattern of the universal micromachining platform includes:

- a machining table with workpiece fixture, freely positionable in three axes
- a tool magazine, freely positionable in one axis, equipped with
  ○ Processing optics 1: Mask projection system,
  ○ Processing optics 2: Two-beam interference system,
  ○ canner: Raylase MINISCAN-7,
  ○ Microscope camera.
- Beam source magazine, freely positionable in an axis, equipped with
  ○ Laser 1: UV short pulse laser TECH-263 (Laser Export Co. Ltd),
  ○ Laser 2: e.g., UV short pulse laser Quanta-Ray Pro290 (Spectra Physics) or Fuego (Time-Bandwidth Products).

All components of the universal micromachining platform (axes, laser, and scanner) are controlled by an embedded system (Zynq™-7000 Development Board, Digilent Inc., Pullmann, WA, USA) with Linux operating system and a tailor-made software. Scanner is controlled via XY2-100 protocol, which is generated in real time by on board Field Programmable Gate Array (FPGA) using a tailor-made design.

Because of the desired structures, corresponding contours can be cut out or removed by means of a scanner. The creation of special surface functionalities and product protection features is achieved by introducing periodic structures using DLIP methods.

### 3.3. Demonstrator of a Universal Micro Physiological System

**Figure 2:**
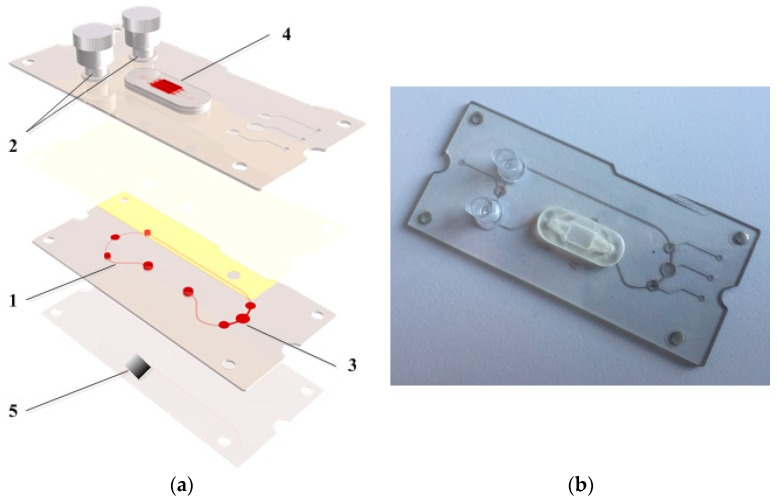
(**a**) Explosion view of the demonstrator system. Closed circulation system: (1) filled with red fluid, consisting of two reservoirs; (2) a three-point-peristaltic-pump; (3) as well as a 3D printed cell culture segment; (4) with integrated chamber and channels. On the bottom is a product protection mark; (5) (**b**) Photograph of the demonstrator.

Based on established microfluidic configurations for substance characterization a demonstrator for the long-term cultivation of scaffolds or tissue slices in a circulation system was conceptualized and successfully realized [4,5,6,7,8,9,10]. Figure 2 shows the explosion view as well as a photo of the working demonstrator. The closed circulation system: (1) filled with red fluid, consisting of two reservoirs; (2) a three-point-peristaltic-pump; (3) as well as a cell culture segment; and (4) The cell culture segment (4) is designed for integrating scaffolds or compact tissue slices. Therefore, a 3D printed polymer construct with integrated chamber and channels is realized. For optimal cell adhesion the surface of the cell culture chamber is hydrophilic and has a periodic line-like structure produced by DLIP. For product protection on the basic chip is marked with a periodic line-like structure with different periods (5).

Subsequent to the fabrication, a complete functionality test of the demonstrator was realized. Besides the leak-proof of the system, the volume flow rates inside the system were characterized by means of Micro-Particle Image Velocimetry [7,24] and pressure ratios by analyzing the deflection of the integrated membranes. It was possible to realize the same fluidic boundary conditions in the demonstrator as in the already established MPS. Initial investigations of biocompatibility and cell culture properties were realized. After coating the microfluidic with fibronectin it was also possible to cultivate cells for several days in the demonstrator and they showed the same adhesion behavior as in the already established MPS. 

Using the universal micromachining platform the realization of MPS including 3D printed components as well as direct laser interference patterned surfaces for well-defined cell adhesion and product protection is possible.

### 3.4. Laser Micro Structuring of Polycarbonate

A Lambda 900 (Perkin Elmer, Beaconsfield, UK) was used to analyze the optical properties (UV-VIS) of the PC foil. Transmittance measurements in the UV-VIS range were carried out under 0° incidence or measurement angle to the substrate normal. Reflection measurements were carried out under an incident or reflection angle of 8° to the substrate normal. The 250 micrometer thick PC foil absorbs up to 95% of the incident irradiation in the wavelength range of 200–380 nm. Due to this optical properties PC foils can be structured and cut with laser irradiation in this wavelength range.

Subsequent the results of the determination of ablation threshold and ns-DLIP carried out with the Quanta-Ray Pro290 laser will be shown exemplary. 

Micro structuring of PC requires the knowledge of the ablation threshold of the substrate material PC at the used laser wavelength. Therefore, ablation tests were realized. The ablation threshold fluence for 266 nm wavelength for PC was determined at 0.05 J·cm^−2^. Higher fluences show an ionization of water molecule in air that result in a plasma shielding effect. Incoming laser irradiation is partially absorbed by the formed plasma. Hence, the ablation threshold rises in the case of plasma shielding to values above 0.44 J·cm^−2^. 

**Figure 3:**
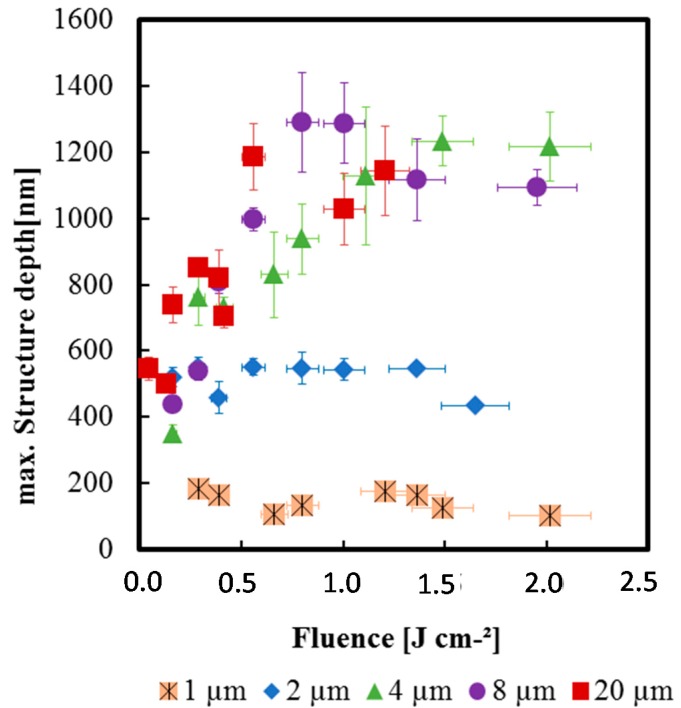
Exemplary dependence on maximum structure depth and fluence for line-like structures at PC (266 nm, 10 ns) with different periods.

Generating product protection features and targeted functionalization of the internal channels, the DLIP process has been successfully established for structuring the PC surfaces. Hence, patterning is performed on the absorbent material by e.g., heated, melted or removed. Using the DLIP method, periodic line structures (in the form of cavities) having a 0.7 to 20 μm period at PC could be generated using a short-pulse UV laser at 266 nm wavelengths. Figure 3 shows exemplary the dependence on maximum structure depth from laser fluence for line-like structures at PC with different periods. Using fluences below 0.1 J·cm^−2^, the interaction of laser radiation with PC generates surface modifications (possible changes on material structure, e.g., refractive index) but no periodical structures. Well-defined, sinusoidal line-like structures with periods between 0.7 and 10 μm were produced with fluences between 0.1–0.8 J·cm^−2^. For fluences above 0.8 J·cm^−2^, strong material melting goes also the formation of superstructures, while the period of the line structure was maintained. The melting of the material increased with increasing pulse number. Line-like structures with a homogeneous, sinusoidal line-like geometry with the avoidance of superstructures were formed at *F* = 0.4–0.8 J·cm^−2^. Similar results were also observed using the TECH-263 laser.

**Figure 4:**
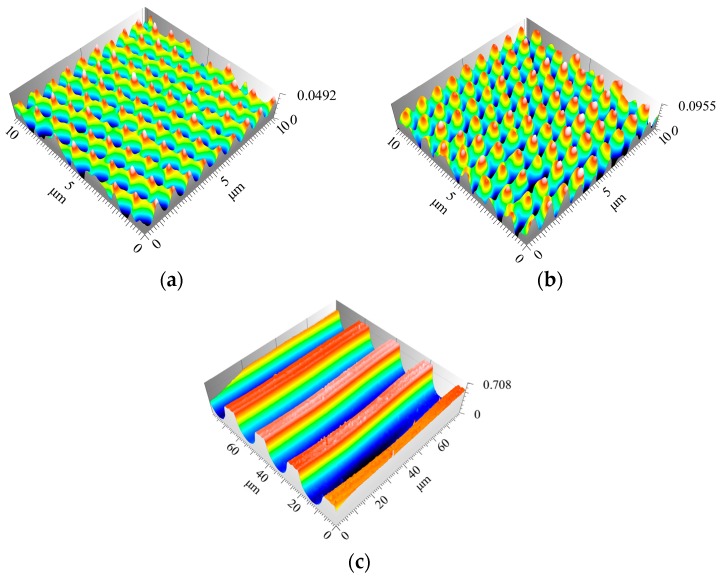
Topography pictures of possible DLIP-Microstructures at 266 nm (ns). (**a**) Two-beam-DLIP; 1 µm periodic cross-like structure (**b**) Three-beam-DLIP; 1.2 µm periodic burl like structure. (**c**) Two-beam-DLIP; 20 µm periodic line-like structure.

Amongst others, structures shown in Figure 4 are generated over a large area by means of ns-DLIP. 

### 3.5. Plastic Freeforming of Microfluidic Components

The PF-process enables the successful construction of application-specific reservoirs and cell structure segments directly on the basic chips. In the first investigations, the cell culture reservoirs were manufactured from the copolymer ABS. The focus was on the optimization of the process parameter concerning the fluid tightness and the bonding on the basic chip made of PC. Moreover, it is intended to avoid support material. This way it can be ensured that no residues of the water soluble or non-biocompatible material remain in the system. A design adjustment of the inner structure minimizes the floating overhangs in the range of the flow channel. Due to this adjustment, the use of any kind of support material can be avoided. 

Apart from avoiding support material, the aim was to apply the cell culture reservoir on the basic chip without the need for any adhesives. In the PF-process, basic chips can be inserted into the build chamber and be printed directly. The deposition of the molten polymer droplets on the thermoplastic basic chips is similar to the welding process of polymers. At the beginning of the manufacturing process, the basic chip in the tempered build chamber is preheated. The deposited molten droplets selectively fuse the basic chip so that a compound is formed on a molecular level. 

The cell culture reservoirs have the purpose to absorb, store and pass the microfluidic into the MPS. Therefore, the tightness of the whole system is crucial to ensure the functionality. Due to the process principle of the freeformer the generated components have a porous structure. The structure is generated by applying single polymer droplets, so that cavities are formed between the droplets. The optimization of the process parameters aimed to minimize the porosity of the cell culture reservoirs to ensure the fluid tightness. 

#### Optimization of the Process Parameters

**Figure 5:**
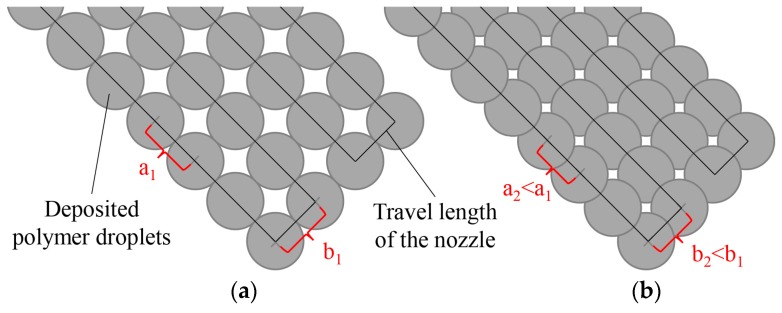
Visualization of the form factor: large form factor (**a**), small form factor (**b**).

The cell culture reservoirs were produced with a 0.2 mm nozzle and the layer thickness will be 0.15 mm. This enables an accurate generation of fine structures. By adjusting the form factor (FF), the degree of filling and thus the pore volume can be varied. This parameter describes the ratio between the width and the height of the droplet (W/H). Figure 5 schematically shows the impact of the differently sized form factors on the filling degree of the component. 

Due to the decrease of the form factor, the gap between the droplets (a*_i_*) as well as the gap between the deposited droplet chains (b*_i_*) is reduced. A too low form factor results in overfilled components. An overfill has a negative effect on the resulting surface quality and leads to an abortion of the building process. The surface quality was not taken into account during this investigation. The focus was on the reduction of the pore volume and the generation of a fluid tight cell culture reservoir. 

Besides the impact of the form factor, the impact of the processing temperature (material preparation and build chamber temperature) is investigated as well. Those process parameters affect the mass temperature of the molten polymer droplets. A temperature increase results in a decrease of the viscosity [18]. Expectably, a decrease of the viscosity improves the wettability of the droplets, so that less cavities are generated. The lower viscosity, therefore, is expected to result in a reduction of the pore volume. The setting behavior of the polymer droplets immediately after the deposition is mainly affected by the temperature of the build chamber. It is expected that a high temperature in the build chamber slow down the setting process. The positive effect of a slower solidification is a better wetting of the previously generated component structure. 

**Figure 6:**
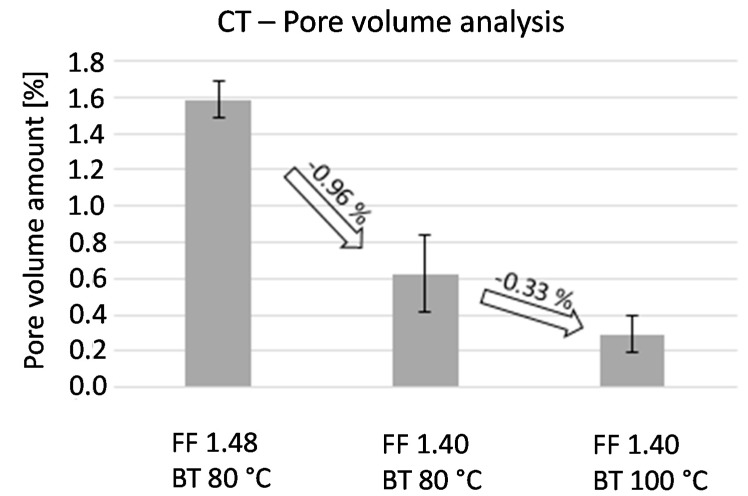
Impact of the form factor (FF) and the build chamber temperature (BT) on the pore volume percentage of the cell culture reservoirs using CT.

The analysis of the pore volume was conducted with the computer tomography system phoenix nanotom m of General Electrics. Figure 6 graphically sums up the quantitative evaluation of the pore volume analysis. 

The diagram shows that the pore volume percentage is 1.6% at a form factor of 1.48 and a build chamber temperature of 80 °C (default values). Owing to a decrease of the form factor on the value of 1.4, the pore volume factor can be reduced by 0.96%. A further reduction to a pore volume of 0.3% can be realized by an increase of the temperature in the build chamber to 100 °C. The machine limits the maximum adjustable temperature in the build chamber, so a further increase of the temperature above 100 °C is not possible now. 

**Figure 7:**
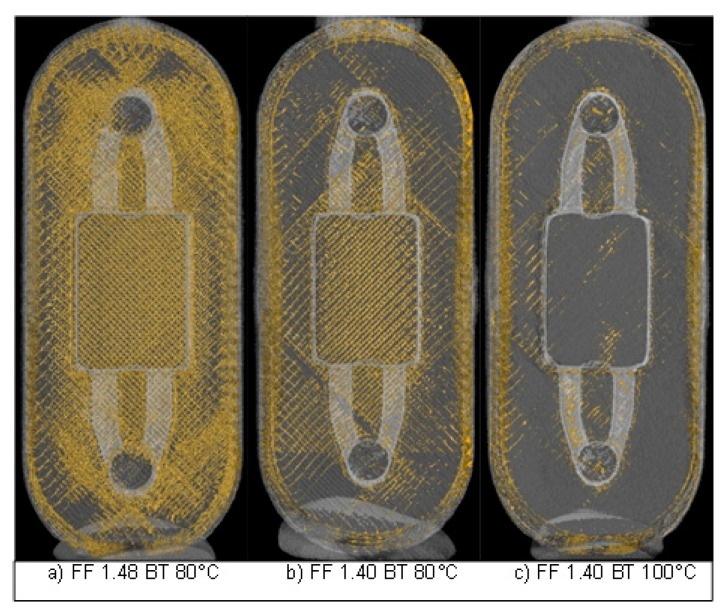
Computer tomographic pore volume analysis with varied form factors (FF) and build chamber temperatures (BT): (**a**) FF 1.48 BT 80 °C; (**b**) FF 1.40 BT 80 °C; (**c**) FF 1.40 BT 100 °C.

Figure 7 shows the three-dimensional view of three cell culture reservoirs produced with the process parameters from Figure 6. The yellow colored areas mark the pores in the test samples. The integrated structures are clearly recognizable in the middle of the figure. Those cavities are not considered in the pore volume analysis. It is clear to see that a low form factor and a high temperature in the build chamber result in a decrease of the pore volume. 

An impact of the cylinder or nozzle temperature on the pore volume could not be proved. The joint between the basic platform and the cell culture reservoir is fluid tight and thereby satisfying. Therefore, it fulfills the given requirements.

## 4. Conclusions and Outlook

For fast and flexible manufacturing and marking of MPS, a concept for a universal micromachining platform has been developed which provides the following latest key technologies: laser micro cutting of polymer foils, laser micro- and sub-micro-structuring of polymer foils, 3D printing of polymer components, as well as optical inspection and online process control. The combination of different laser sources, processing optics, inspection systems, and print heads on multiple axes allows the change and exactly positioning to the workpiece during the process with an accuracy of less than 2 micrometers. Therewith the realization of MPS including 3D printed components as well as direct laser interference patterned surfaces for well-defined cell adhesion and product protection is possible.

A basic technology for the generation of periodical line-like structures with periods between 0.7–20 µm at PC foil using special DLIP-optic in combination with a laser source (wavelength 263–266 nm, ns) was established.

Furthermore, the investigations in this paper have shown that fluid-tight cell culture reservoirs made of ABS can be built directly onto raw basic chips. 

A first prototype of the universal micromachining platform combining different lasers with DLW- and DLIP-modules was established. With this laser micro cutting, as well as laser micro-structuring of PC foils, and therewith functionalization for the MPS application could be shown successfully. 

In further studies, it is intended to conduct a material qualification of PC for the PF-process. The aim is to generate the cell culture reservoirs made of PC directly on the basic chips. The major advantage of PC is that it is qualified for cell cultivation and does not have any toxic effect on the cells. Compared to ABS, it is also more transparent so the cell culture compartments can be inspected visually by using microscopy. The weldability of thermoplastic polymers is dependent on their compatibility. Therefore, in the welding of similar polymers higher joint strengths can be achieved. By using a PC material for the cell culture reservoirs, the chemical bonding to the basic chip, consisting of PC as well, can be improved. The processing of PC requires higher temperatures in the build chamber. This temperature increase supports the welding process between the applied droplets and the basic platform additionally. The exact positioning of the cell culture reservoir on the basic chip was not prioritized until now but will be a part in further investigations. A methodology shall be developed to realize a defined and reproducible positioning of the cell culture reservoirs on the basic chips.

In further work, the closed machine system freeformer is to be integrated into the process chain and the structure of the micromachining platform. This can be realized by positioning the basic chip as an insert in the installation space manually or with the aid of a robot system. Subsequently, the application-specific reservoirs and cell culture segments are built on the basic chip in combination with a directly printed gasket. The newly formed composite structure is removed and fed to the further processing stations. 

In addition to the already integrated online monitoring methods (microscope, power measurement), further methods (temperature, beam profile) are to be integrated into the process.
